# Yves Sintomer, 2016. Das demokratische Experiment. Geschichte des Losverfahrens in der Politik von Athen bis heute: Wiesbaden: VS Springer Verlag. 281 S., 978-3658007300, 49,99 € (Print, Softcover)

**DOI:** 10.1007/s12286-021-00478-5

**Published:** 2021-03-12

**Authors:** Michael Kolkmann

**Affiliations:** Regierungslehre und Policyforschung, Martin-Luther-Universität Halle-Wittenberg, Emil-Abderhalden-Str. 26–27, 06108 Halle (Saale), Deutschland

Seit vielen Jahren wird im öffentlichen wie im wissenschaftlichen Diskurs über die „Krise der Demokratie“ gesprochen, über „Postdemokratie“ und „Postparlamentarismus“. Neben der Krise *der* Demokratie als Konzept wird dabei häufig auf krisenhafte Teilaspekte von modernen Demokratien Bezug genommen: genannt werden in der Regel abnehmende Zustimmung zu Formen repräsentativer Demokratie in der Bevölkerung, rückläufige Parteimitgliedschaftszahlen, sinkende Wahlbeteiligung, augenscheinlich zunehmende Politikverdrossenheit. Auf unterschiedlichen politischen Ebenen werden seit geraumer Zeit neue innovative Beteiligungsformen von Bürgerinnen und Bürgern ausprobiert. So begleitet aktuell in Frankreich ein Bürgerrat die Impfkampagne der Regierung („collectif de citoyens“), in Thüringen wird nach Beschluss der Landesregierung von Anfang Februar 2021 zur Begleitung der Corona-Krise ein „Thüringer BürgerForum“ gegründet, was es in ähnlicher, wenn auch deutlich kleinerer Form in der Stadt Augsburg bereits gibt. Ähnliche Runden kennen etwa die französische Stadt Grenoble („Comité de liaison citoyen COVID-19“) oder der US-Bundesstaat Oregon („Oregon Citizens’ Assembly on Covid-19 Recovery“).

Gemeinsam ist diesen Formaten, dass ihre Zusammensetzung gelost wird, und zwar in der Regel auf Basis von Geschlecht, Alter, Bildungsabschluss und Migrationshintergrund. Als die damalige Landesregierung von Rheinland-Pfalz in den 1990er-Jahren entschied, bei uneinigen Fragen im Bundesrat eher den Losentscheid (offiziell: „Wechselstimmverfahren“) zu nutzen, als sich zu enthalten (Stimmenthaltungen werden im Bundesrat als Nein-Stimmen gewertet), wurde diese Entscheidung öffentlich vor allem negativ kommentiert. Dabei handelt es sich beim Losverfahren um eine uralte Möglichkeit, zu politischen Entscheidungen zu kommen, wie der französische Politikwissenschaftler *Yves Sintomer* in seinem Buch „Das demokratische Experiment“ hervorhebt. Darin spannt er den Bogen von ersten Versuchen in der Antike bis hin zu zeitgenössischen Beispielen aus der aktuellen politischen Praxis.

Erstmals im Jahr 2007 unter dem Titel „Le pouvoir au peuple“ („Die Macht dem Volke“) erschienen, wurde das Buch für seine deutschsprachige Fassung aus dem Jahre 2016 aktualisiert und erweitert, auch wenn die grundlegenden Thesen seiner Argumentation, wie Sintomer betont, gleichgeblieben sind. Ausgangspunkt für die erste (französische) Ausgabe waren Vorschläge der damaligen Präsidentschaftskandidatin Ségolène Royal, so genannte „Bürgerjurys“ einzurichten: diese „hatten so heftige Reaktionen hervorgerufen, dass es die Mühe zu lohnen schien, die Gründe für die Heftigkeit jener Debatte auszuloten, zu ihr beizutragen, indem man einen soziologischen und historischen Abstand einnimmt und diesen Faden aufgreift für eine systematische Analyse von Erfahrungen mit dem Losverfahren in der Politik“ (S. 249).

Yves Sintomer zeigt in seinem Werk Möglichkeiten partizipativer, deliberativer beziehungsweise radikaler Demokratie auf, denen gemeinsam ist, dass die Bürgerinnen und Bürger entscheidenden Einfluss auf die Prozesse der politischen Entscheidungsfindung nehmen können. Das Ziel des Buches formuliert der Autor wie folgt: „Wir müssen uns ohne Vorurteile die Frage stellen: Öffnet die Idee, das Losverfahren wiedereinzuführen, einen vielversprechenden Weg für die heutigen Demokratien? Könnten solche partizipativen Instanzen eine Quelle der Demokratisierung bilden, einen Ausgangspunkt für eine aufgeklärtere öffentliche Meinung und ein verantwortlicheres politisches Handeln, kurz: für eine Dynamik, die dem ‚Populismus‘ und der ‚Mediendemokratie‘ gerade entgegenwirkt? Welche Bedingungen müssten erfüllt sein? Was wären die Herausforderungen, denen man sich stellen müsste?“ (S. 6).

Angeleitet wird die Analyse des Autors durch vor allem drei zentrale Fragen, die er im Verlaufe seiner Argumentation immer wieder aufgreift und am Ende zusammenführt: „1) Welche Bedeutungen hatte die politische Verwendung des Losverfahrens in der Antike, im Mittelalter oder in der Renaissance? Können wir in der Nachfolge alter (wie Aristoteles, Leonardo Bruni und Francesco Guicciardini) und zeitgenössischer Autoren (wie Bernard Manin oder Jacques Rancière) festhalten, dass das Losverfahren mit der Demokratie eng zusammenhing? 2) Warum ist das Losverfahren von der politischen Bühne mit den modernen Revolutionen verschwunden, während es gleichzeitig in den Geschworenengerichten der Rechtsprechung häufiger wurde? 3) Was bedeuten seine heutige Rückkehr in zahlreichen Modellen und deren exponentielle Vervielfältigung?“ (S. 250).

Im zweiten Kapitel „Eine nicht enden wollende Krise der Repräsentation“ lotet Sintomer zunächst jene Legitimationskrise aus, die er in Bezug auf das politische System diagnostiziert. In diesem Abschnitt wird eine der zentralen Aussagen seines Buches formuliert: „Die heutigen Demokratien stehen einem Paradox gegenüber. Auf der einen Seite war das demokratische Regime noch nie so weit über den Planeten verbreitet, und die Ereignisse der 2010er Jahre bestätigen seine Attraktivität für Bevölkerungen, die unter autoritären Regimen leben. Auf der anderen Seite leiden die repräsentativen Regierungen unter einer schleichenden Legitimitätskrise, werden von der Globalisierung und der Krise eines Wirtschaftsmodells erschüttert, das auf der Hegemonie des Finanzkapitals aufbaut, und scheinen nicht in der Lage zu sein, die ökologischen Herausforderungen zu meistern. Während sich der soziale Wandel beschleunigt, finden die wesentlichen demokratischen Innovationen fern einer weitgehend erstarrten institutionellen Politik statt“ (S. 9). Die perzipierte Krise der Repräsentation operationalisiert Sintomer anhand von sechs strukturellen Merkmalen: die „machtlose Politik“, der „politische Niedergang der unteren Klassen“, die „Entstehung der Risikogesellschaft“, die „Krise des bürokratischen öffentlichen Handelns“, die „ideologische Hürde“ und „Ursachen im Inneren des politischen Systems“ (vgl. S. 12 ff.).

Anschließend geht Sintomer im dritten Kapitel („Das Losverfahren in der Geschichte: Die Zähmung des Zufalls?“) historisch gesehen weit zurück, um nachvollziehen zu können, wie die Verwendung des Losverfahrens, das in den antiken Demokratien und den italienischen Stadtrepubliken eine entscheidende Rolle gespielt hat, in den modernen Demokratien auf Geschworenenjurys beschränkt wurde: „über lange Zeit ist die Auslosung ein konstitutiver Bestandteil demokratischer und republikanischer Systeme gewesen“ (S. 31). Dieses Kapitel ist sehr breit aufgestellt: berücksichtigt werden Vorderasien, Athen, Rom, einige italienische Republiken (etwa Venedig und Florenz) sowie Spanien. Dieses Kapitel ist nicht zufällig das ausführlichste des gesamten Buches. Diesen historischen Exkurs summiert Sintomer zu drei denkbaren Anwendungsmöglichkeiten: „1. Das Losverfahren kann in der Politik eine übernatürliche oder religiöse Dimension haben, wenn es dazu dient, den göttlichen Willen auszudrucken […]. 2. Es ist darüber hinaus ein unparteiisches Instrument zur Konfliktlösung, insbesondere beim Wettbewerb um Machtpositionen. 3. Schließlich garantiert es Chancengleichheit beim Zugang zu politischen oder judikativen Ämtern und fordert die Selbstregierung der Bürger […]. Es unterscheidet sich also von erblichen Übertragungsmechanismen von Macht, wie sie für Monarchien typisch sind, aber auch von der Kooptation von oben, der Ernennung durch übergeordnete Behörden, dem Ämterkauf und schließlich der Wahl“ (S. 104). Beendet wird dieses Kapitel mit Hilfe eines Rückgriffs auf Bernard Manin mit der Frage, warum das Los als politisches Instrument verschwunden ist (vgl. S. 104 ff.).

In Kapitel fünf wendet sich der Autor konkreten Fallbeispielen zu, und das auf globaler Ebene. Wo gibt es solche Verfahren, wie sind sie strukturiert, wer ist beteiligt und wie schauen die konkreten Ergebnisse dieser Verfahren aus? Hier spannt Sintomer den Bogen von den Vereinigten Staaten und Dänemark bis hin zu Japan und Großbritannien, von der Meinungsforschung bis hin zu Bürgerhaushalten, von Bürgerversammlungen bis zu Konsenskonferenzen.

Im sechsten Kapitel („Die Demokratie erneuern“) werden die vorgestellten Formen als Alternative zu politischen Parteien und deren Bedeutungsverlust diskutiert. In analytischer Hinsicht unterscheidet Sintomer fünf große Modelle politischer Logiken: 1. Divination. Das Losverfahren kann demnach in einer religiösen oder übernatürlichen Perspektive interpretiert werden. 2. Unparteilichkeit. Die Zufallsauswahl lässt sich als eine unparteiische Methode betrachten, um eine umstrittene Frage zu klären. 3. Selbstregierung bzw. radikale Demokratie. Das Losverfahren kann außerdem als ein Verfahren begriffen werden, das eine Selbstregierung aller durch alle ermöglicht, indem jeder abwechselnd Regierter und Regierender ist. 4. Gesunder Menschenverstand der Laien. Auf etwas andere Weise lässt sich sagen, dass das Losverfahren sicherstellt, dass die Macht über alle von jedem ausgeübt wird, das heißt von austauschbaren Individuen, soweit sie über „gesunden Menschenverstand“ verfügen. 5. Deliberative Demokratie. Diese Verfahren zeichnen sich wiederum dadurch aus, dass sie zunächst das Losverfahren als ein Mittel betrachten, eine repräsentative Stichprobe zu erstellen, eine Art Mikrokosmos der politischen Gemeinschaft, die da zu begutachten, evaluieren, beurteilen und gelegentlich im Namen der Gemeinschaft zu entscheiden vermag, wo nicht alle an der Deliberation teilnehmen können (vgl. als Zusammenfassung insbesondere die Tabelle auf S. 211).

Was das Buch über die erwähnten, hier knapp referierten Aspekte hinaus charakterisiert, ist, dass es dazu einlädt, „die Grundfragen jeder demokratischen Ordnung neu zu stellen: Welche sind die Quellen politischer Legitimität? Wer entscheidet über und was bedeutet konkret Volkssouveränität? Was ist der Sinn der Repräsentation? Wie lässt sich das allgemeine Interesse kollektiv verhandeln und konstruieren?“ (S. 7). Für eine systematische Grundlegung der Analyse heutiger Formate, wie sie eingangs erwähnt wurden, bietet das Buch eine vorzügliche Grundlage. Gleichwohl wäre es mit Blick auf eine breite Debatte zum Thema wünschenswert, diesen Forschungsstrang pointiert und fundiert weiterzuverfolgen. Als Leser dieses (2016 erschienenen) Werkes würde man zum Beispiel gerne erfahren, was Sintomer von der „Grand Débat“ des französischen Präsidenten Emmanuel Macron hält, einer Art Bürgerrat, den Macron auf dem Höhepunkt der Gelbwesten-Bewegung initiiiert hat.

Natürlich wird angesichts des breiten Themenspektrums des Bandes einiges eher holzschnittartig gezeichnet, anderes hätte man sich vertieft gewünscht. Nicht überraschenderweise beziehen sich viele Fallbeispiele und Ausführungen auf das Nachbarland Frankreich, diese werden jedoch ergänzt durch Formate aus anderen europäischen Ländern, nicht zuletzt Deutschland. Teilweise wird in einzelnen Abschnitten zwischen der Rolle von Volksparteien, den Federalist Papers und Begrifflichkeiten wie Publikums- oder Mediendemokratie hin und her gesprungen. Gelegentlich finden sich Allgemeinplätze („Die Beschäftigung mit der Krise der Demokratie ist so alt wie die Demokratie selbst“, S. 24 oder „Eine andere Welt ist möglich“, S. 243). Aber Sintomer präsentiert eine ganze Reihe unterschiedlicher Ansätze, die zum Ein- und Weiterlesen einladen: beeindruckend ist die Breite der vorgestellten Ideen und Autoren von Cicero bis Tocqueville sowie von Hegel bis Habermas; dabei bietet er eine Mischung aus juristischen, politik- und gesellschaftspolitischen und philosophischen Zugängen zum Thema.

Wünschenswert wäre grundsätzlich auch, über die im Buch erwähnten Beispiele hinaus andere Möglichkeiten deliberativer Demokratie in die Debatte einzubeziehen. Der sächsische Ministerpräsident Michael Kretschmer (CDU) schlug etwa vor einiger Zeit einen „Volkseinwand“ vor. Demnach sollten praktisch alle Gesetze des Landtages durch eine Volksabstimmung gekippt werden können. Und ausgerechnet Bundestagspräsident Wolfgang Schäuble (ebenfalls CDU) sieht in Bürgerräten eine Möglichkeit, die Bevölkerung stärker in die Politik einzubinden und erwartet, dass solche Instrumente Vertrauen in die Politik stärken und der repräsentativen Demokratie neue Impulse geben können. Zudem wird seit vielen Jahren die Möglichkeit direktdemokratischer Elemente auch auf Bundesebene (über den bislang einzigen Fall nach Art. 29 Abs. 3 Grundgesetz hinaus) diskutiert, während dieses Instrument auf Landes- wie auf kommunaler Ebene breit genutzt wird.

Und auch die Wechselbeziehung der erwähnten partizipativen Verfahren mit den grundlegenden institutionellen Strukturen des parlamentarischen Systems scheint in Zukunft einen Blick wert zu sein: welche Konsequenzen ergeben sich durch die Anwendung der erwähnten Formate für Verfahren in Parlament und Regierung und nicht zuletzt für das Selbstverständnis von gewählten Volksvertretern? Es scheint, als wurde diese Debatte soeben erst angestoßen. Spannend ist dabei die Frage, ob Mitglieder von Bürgerräten in ihren Entscheidungen freier sind, da sie, anders als Mitglieder von Parlamenten, nicht wiedergewählt werden können bzw. müssen. Andererseits kann thematisiert werden, dass gerade hierin eine spezifische politische Responsivität von gewählten Repräsentanten liegen könnte.

Der Greifswalder Politikwissenschaftler Hubertus Buchstein etwa empfiehlt solche gelosten Gremien – neben anderen Gründen – für die Beseitigung politischer Blockaden bei besonders komplexen Gegenständen, wie etwa einer Reform des bundesdeutschen Wahlrechts. Mit Blick auf das „Reförmchen“ (Dietmar Bartsch) der Großen Koalition aus dem Herbst 2020 böte sich dieses Themenfeld in der Tat als (erstes) Versuchsfeld an. Unabhängig davon, wie sich die konkrete öffentliche wie wissenschaftliche Debatte zu diesem Untersuchungsgegenstand gestalten wird: Yves Sintomer hat mit seinem Buch „Das demokratische Experiment“ eine detaillierte und prägnante systematische Grundlage für die zukünftige Beschäftigung mit allen Spielarten des Losentscheids geliefert.

